# Supplementary material for: Hydrogenation of β-Keto Sulfones to β-Hydroxy Sulfones with Alkyl Aluminum Compounds: Structure of Intermediate Hydroalumination Products
Source: Molecules. 2022 Apr 6;27(7):2357. doi: 10.3390/molecules27072357 (PMC9000326; doi:10.3390/molecules27072357)
Supplement: Supplementary file 1 [file molecules-27-02357-s001.zip › molecules-1636926-supplementary.pdf]

Supplementary Materials

Hydrogenation of β-Keto Sulfones to β-Hydroxy Sulfones with Alkyl Aluminum Compounds: Structure of Intermediate Hydroalumination Products

Michał Kotecki [1], Zbigniew Ochal [1], Paweł Socha [2], Vadim Szejko [1], Łukasz Dobrzycki [2], Mariola Stypik [1] and Wanda Ziemkowska [1,*]

[1] Faculty of Chemistry, Warsaw University of Technology, Noakowskiego 3, 00-664 Warsaw, Poland; michal.kotecki2.stud@pw.edu.pl (M.K.); zbigniew.ochal@pw.edu.pl (Z.O.); vsheiko@ch.pw.edu.pl (V.S.), mariola.stypik.dokt@pw.edu.pl (M.S.)
[2] Department of Chemistry, University of Warsaw, Pasteura 1, 02-093 Warsaw, Poland; psocha@chem.uw.edu.pl (P.S.), dobrzyc@chem.uw.edu.pl (Ł.D.)
* Correspondence: ziemk@ch.pw.edu.pl or wanda.ziemkowska@pw.edu.pl

Table of contents:

Characterization of *β*-keto sulfones **1a-1e**.

Characterization of *β*-hydroxy sulfones **4a-4e**.

Figure S1. ^{1}H NMR spectrum of the compound **2aa** – hydroalumination product of a β-keto sulfone **1a** with *i*-Bu_3Al.

Figure S2. ^{1}H NMR spectrum of the compound **2aa** – expanded part of the spectrum showing *i*-BuAl proton signals.

Figure S3. ^{13}C NMR spectrum of the compound **2aa**.

Figure S4. ^{1}H NMR spectrum of the compound **2ab** – hydroalumination product of a β-keto sulfone **1b** with *i*-Bu_3Al.

Figure S5. ^{1}H NMR spectrum of the compound **2ab** – expanded part of the spectrum showing *i*-BuAl proton signals.

Figure S6. ^{13}C NMR spectrum of the compound **2ab**

Figure S7. ^{1}H NMR spectrum of the compound **2ba** – hydroalumination product of a β-keto sulfone **1a** with Et_3Al (1:1).

Figure S8. ^{1}H NMR spectrum of the compound **2ba** – expanded part of the spectrum showing EtAl proton signals.

Figure S9. ^{13}C NMR spectrum of the compound **2ba**.

Figure S10. ^{1}H NMR spectrum of the compound **2bb** – hydroalumination product of a β-keto sulfone **1b** with Et_3Al (1:1).

Figure S11. ^{1}H NMR spectrum of the compound **2bb** – expanded part of the spectrum showing EtAl proton signals.

Figure S12. ^{13}C NMR spectrum of the compound **2bb**.

Figure S13. ^{1}H NMR spectrum of the compound **3bb** – hydroalumination product of a β-keto sulfone **1b** with Et_3Al (1:2).

Figure S14. ^{1}H NMR spectrum of the compound **3bb** – expanded part of the spectrum showing EtAl proton signals.

Figure S15. ^{13}C NMR spectrum of the compound **3bb**.

Characterization of *β*-keto sulfones 1a-1e.

2-(*p*-Tolylsulfonyl)acetophenone (**1a**) ^{1}H NMR δ: 7.95 (2H, dd, J_{3H} = 8.2 Hz, J_{4H} = 1.4 Hz, H_{aromat}), 7.77 (2H, d, J_{3H} = 8.2 Hz, H_{aromat}), 7.63 (2H, m, H_{aromat}), 7.49 (3H, m, H_{aromat}), 7.34 (2H, d, J_{3H} = 7.6 Hz, H_{aromat}), 4.72 (2H, s, CH_2), 2.45 (3H, s, CH_3Ph). ^{13}C NMR δ: 192.43 (C=O), 141.16, 135.91, 134.28, 134.17, 131.32, 129.20, 129.14, 128.84 (C_{aromat}), 64.94 (CH_2), 13.32 (CH_3Ph) ppm. Mp.: 135-136ºC.

2-(*p*-Chlorosulfonyl)acetophenone (**1b**) ^{1}H NMR δ: 7.93 (2H, dd, J_{3H} = 8.5 Hz, J_{4H} = 1.4 Hz, H_{aromat}), 7.83 (2H, d, J_{3H} = 8.5 Hz, H_{aromat}), 7.64 (1H, m, H_{aromat}), 7.53-7.47 (4H, m, H_{aromat}), 4.75 (2H, s, CH_2). ^{13}C NMR δ: 187.87 (C=O), 141.08, 136.93, 135.46, 134.54, 130.14, 129.49, 129.21, 128.91 (C_{aromat}), 63.23 (CH_2) ppm. Mp.: 107-108ºC.

2-(*p*-Tolylsulfonyl)acetone (**1c**) ^{1}H NMR δ: 7.75 (2H, d, J_{3H} = 8.0 Hz, H_{aromat}), 7.36 (2H, d, J_{3H} = 8.0 Hz, H_{aromat}), 4.13 (2H, s, CH_2), 2.44 (3H, s, CH_3Ph). ^{13}C NMR δ: 196.14 (C=O), 145,50, 135,58, 129,99 128,19 (C_{aromat}), 67,83 (CH_2) i 31,47 (C(=O)$\underline{C}H_3$), 21,68 ($PhCH_3$) ppm. Mp.: 56-57°C.

2-(*p*-Tolylsulfonyl)-2-(phenyl)acetophenone (**1d**) ^{1}H NMR δ: 7.88 (2H, d, J_{3H} = 7.3 Hz, H_{aromat}), 7.52-7.19 (12H, m, H_{aromat}), 6.13 (H, s, CH), 2.40 (3H, s, CH_3Ph). ^{13}C NMR δ: 190.75 (C=O), 145.00, 135.99, 133.91, 133.79, 130.38, 130.30, 129.56, 128.99, 128.83, 128.79, 128.73, 128.59 (C_{aromat}), 76.05 (CH_2), 21.68 ($\underline{C}H_3Ph$) ppm. Mp.: 150-154ºC.

2-(*p*-Tolylsulfonyl)-2-(methyl)acetophenone (**1e**) ^{1}H NMR δ: 7.96 (2H, d, J_{3H} = 7.8 Hz, H_{aromat}), 7.64 (2H, d, J_{3H} = 7.8 Hz, H_{aromat}), 7.59 (1H, m, H_{aromat}), 7.46 (2H, m, H_{aromat}), 7.29 (2H, d, J_{3H} = 7.7 Hz, H_{aromat}), 5.16 (1H, q, J_{3H} = 6.7 Hz, C($\underline{H}$)CH_3), 2.41 (3H, s, CH_3Ph), 1.54 (3H, d, J_{3H} = 6.7 Hz, C(H)C$\underline{H}_3$). ^{13}C NMR δ: 192.59 (C=O), 145,34, 136.17, 134.00, 132.95, 129.75, 129.53, 129.14, 128.80 (C_{aromat}), 64.86 ($\underline{C}$(H)CH_3), 21.67 ($\underline{C}H_3Ph$), 13.17 (C(H)$\underline{C}H_3$) ppm. Mp.: 103-105°C.

Characterization of *β*-hydroxy sulfones 4a-4e.

2-((4-Methylphenyl)sulfonyl)-1-phenylethanol (**4a**) ^{1}H NMR δ: 7.81 (2H, d, *J* = 8.2 Hz H_{aromat}), 7.38 (2H, d, *J* = 8.2 Hz, H_{aromat}), 7.29 (5H, m, H_{aromat}), 5.25 (1H, d, *J* = 10.4 Hz, $CH_2C\underline{H}(OH)$), 3.74 (1H, s, $O\underline{H}$), 3.48 (1H, dd, *J* =14.2, 10.2 CH_2), 3.32 (1H, dd, *J* = 14.2, 1.3, CH_2), 2.46 (3H, s, CH_3Ph). ^{13}C NMR δ: 145.25, 140.61, 136.05, 130.08, 128.72, 128.28, 127.98, 125.62 (C_{aromat}), 68.43 (SO_2CH_2), 63.94 (COH), 21.68 (CH_3Ph) ppm. Mp.: 74-75°C.

2-((4-Chlorophenyl)sulfonyl)-1-phenylethanol (**4b**) ^{1}H NMR δ: 7.88 (2H, d, J_{3H} = 8.2 Hz H_{aromat}), 7.55 (2H, d, J_{3H} = 8.2 Hz, H_{aromat}), 7.30 (5H, m, H_{aromat}), 5.28 (1H, d, J_{3H} = 10.4 Hz, $CH_2C\underline{H}(OH)$), 3.55-3.32 (3H, m, $O\underline{H}$, $C\underline{H}_2CH(OH)$). ^{13}C NMR δ: 140.87, 140.45, 137.69, 129.73, 129.53, 128.83, 128.48, 125.62 (C_{aromat}), 68.55 ($\underline{C}H_2CH(OH)$), 63.93 ($CH_2\underline{C}H(OH)$) ppm. Mp.: 105-107°C.

1-((4-methylphenyl)sulfonyl)-2-propanol (**4c**) ^{1}H NMR δ: 7.79 (2H, d, J_{3H} = 8.0 Hz H_{aromat}), 7.37 (2H, d, J_{3H} = 8.0 Hz, H_{aromat}), 4.29 (1H, m, $CH_2C\underline{H}(OH)$), 3.46 (1H, s, broad, OH), 3.23-3.11 (2H, m, $C\underline{H}_2CH(OH)$), 2.45 (3H, s, $C\underline{H}_3Ph$), 1.22 (3H, d, J_{3H} 6.2 Hz, $C(OH)C\underline{H}_3$). ^{13}C NMR δ: 145.22, 136.00, 130.06, 127.92 (C_{aromat}), 63.31 ($SO_2\underline{C}H_2$), 62.33 ($CH_2\underline{C}H(OH)$), 22.48 ($CH(OH)\underline{C}H_3$), 21.66 ($Ph\underline{C}H_3$) ppm. Mp.: 56-57°C.

2-((4-Methylphenyl)sulfonyl)(2-phenyl)-1-phenylethanol (**4d**) ^{1}H NMR δ: 7.51-6.89 (14H, m, H_{aromat}), 5.73 (1H, d, J_{3H} = 9.0 Hz, $C(\underline{H})OH$), 4.68 (1H, s, $O\underline{H}$), 4.43 (1H, d, J_{3H} = 9.0 Hz, $C(\underline{H})Ph$), 2.37 (3H, s, $C\underline{H}_3Ph$). ^{13}C NMR δ: 144.99, 139.48, 134.41, 130.28, 129.34, 128.92, 128.77, 128.45, 128.10, 128.06, 128.03, 127.38 (C_{aromat}), 77.40 ($\underline{C}(H)Ph$), 73.82 ($\underline{C}(H)OH$), 21.65 ($\underline{C}H_3Ph$) ppm. Mp.: 159-160 °C.

2-((4-Methylphenyl)sulfonyl)(2-methyl)-1-phenylethanol (**4e**) ^{1}H NMR δ: 7.83 (2H, d, J_{3H} = 8.0 Hz, H_{aromat}), 7.40 (2H, d, J_{3H} = 8.0 Hz H_{aromat}), 6.70 (5H, m, H_{aromat}) 4.91 (1H, d, J_{3H} = 9.1 Hz , $C\underline{H}(OH)$), 4.62 (1H, s, OH), 3.36 (1H, m, $SO_2C\underline{H}$), 2.47 (3H, s, $PhC\underline{H}_3$), 0.80 (3H, d, J_{3H} = 7.1 Hz, $C(H)C\underline{H}_3$). ^{13}C NMR δ: 145.38, 139.58, 133.66, 129.96, 129.08, 128.63, 128.58, 127.09 (C_{aromat}), 73.85 ($SO_2\underline{C}(H)CH_3$), 65.97 ($\underline{C}H(OH)$), 21.71 (($Ph\underline{C}H_3$), 12.96 ($C(H)\underline{C}H_3$) ppm. Mp.: 190-191 °C.

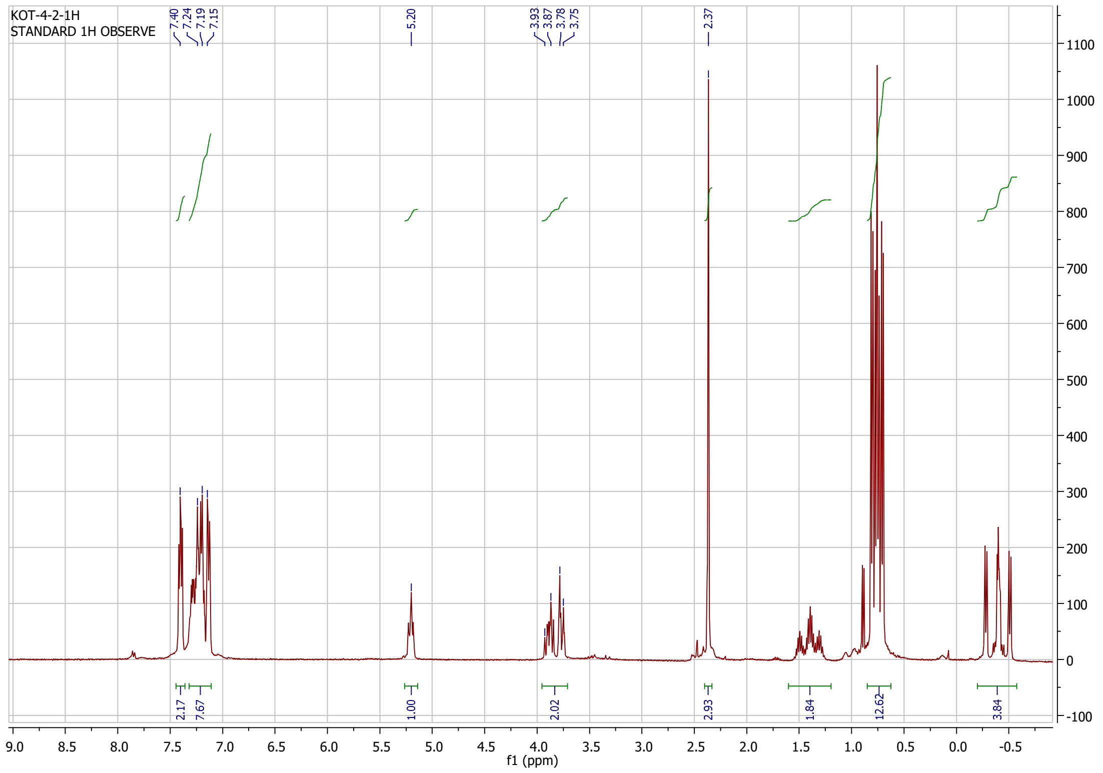

Figure S1. ^{1}H NMR spectrum of the compound **2aa** – hydroalumination product of a *β*-keto sulfone **1a** with *i*-Bu_3Al.

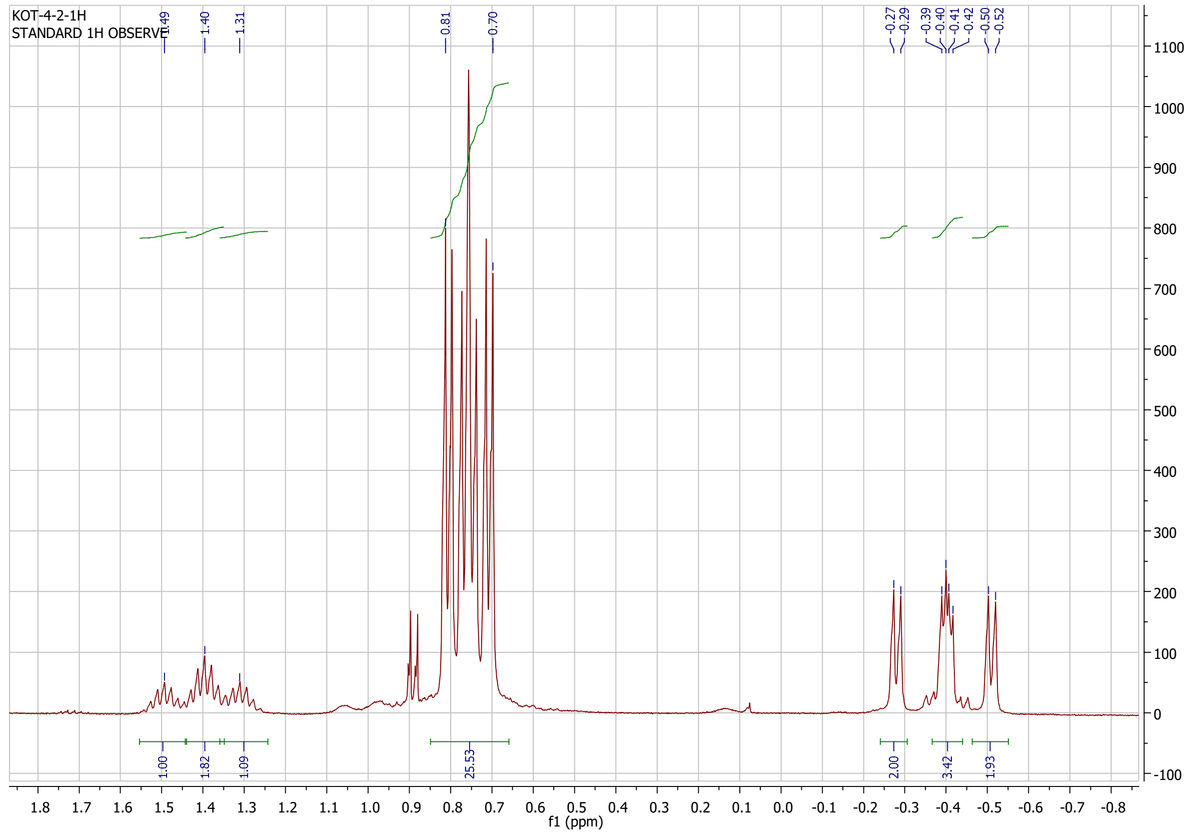

Figure S2. ^{1}H NMR spectrum of the compound **2aa** – expanded part of the spectrum showing *i*-BuAl proton signals.

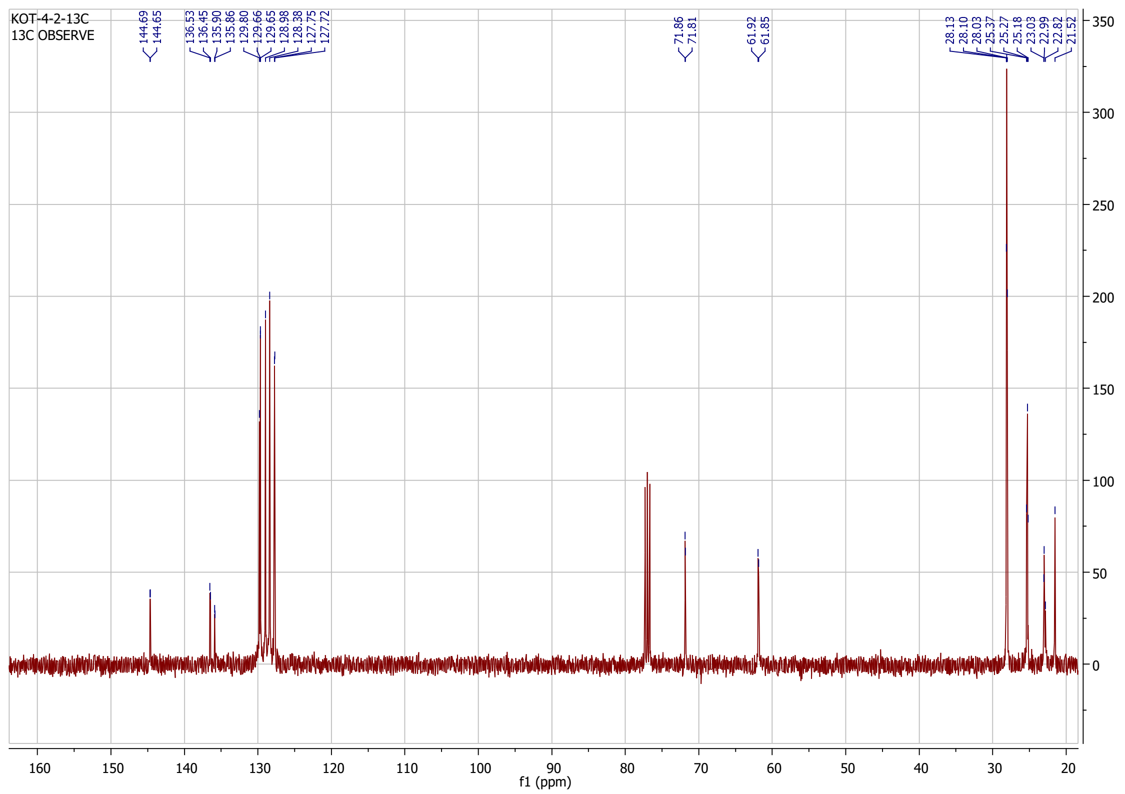

Figure S3. ^{13}C NMR spectrum of the compound **2aa**.

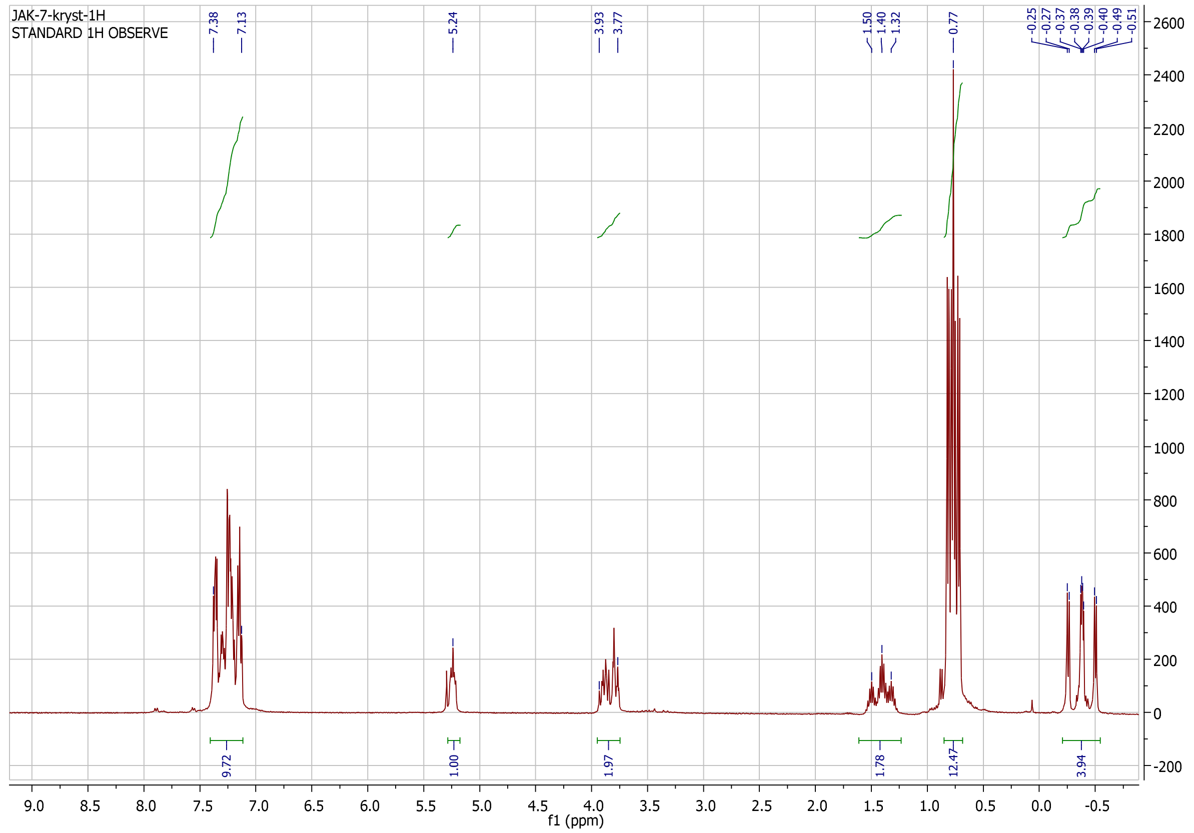

Figure S4. ^{1}H NMR spectrum of the compound **2ab** – hydroalumination product of a *β*-keto sulfone **1b** with *i*-Bu_3Al.

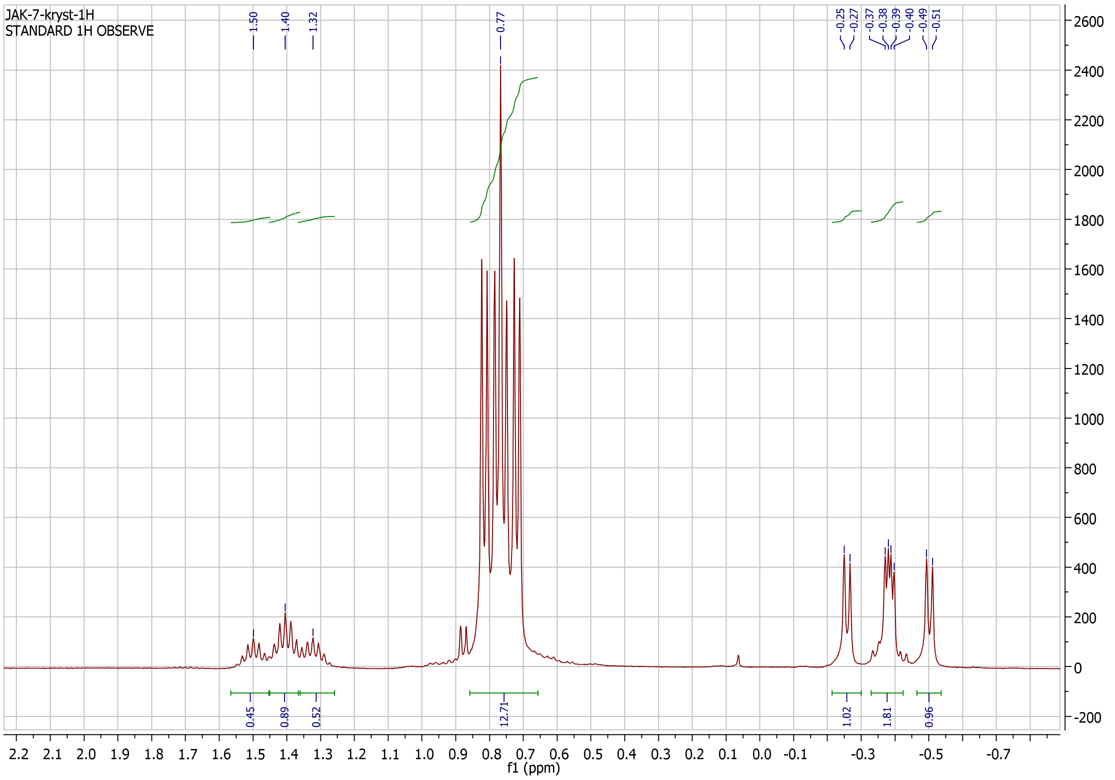

Figure S5. ^{1}H NMR spectrum of the compound **2ab** – expanded part of the spectrum showing *i*-BuAl proton signals.

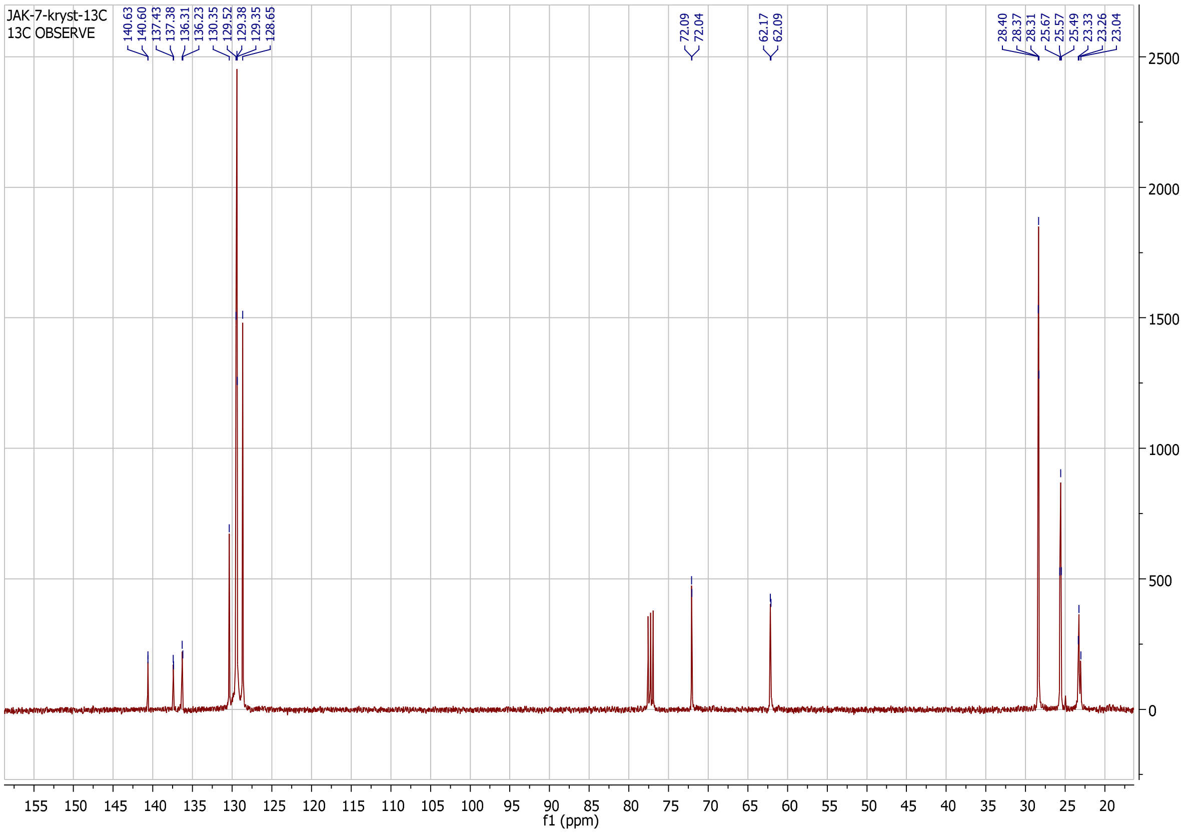

Figure S6. ^{13}C NMR spectrum of the compound **2ab**.

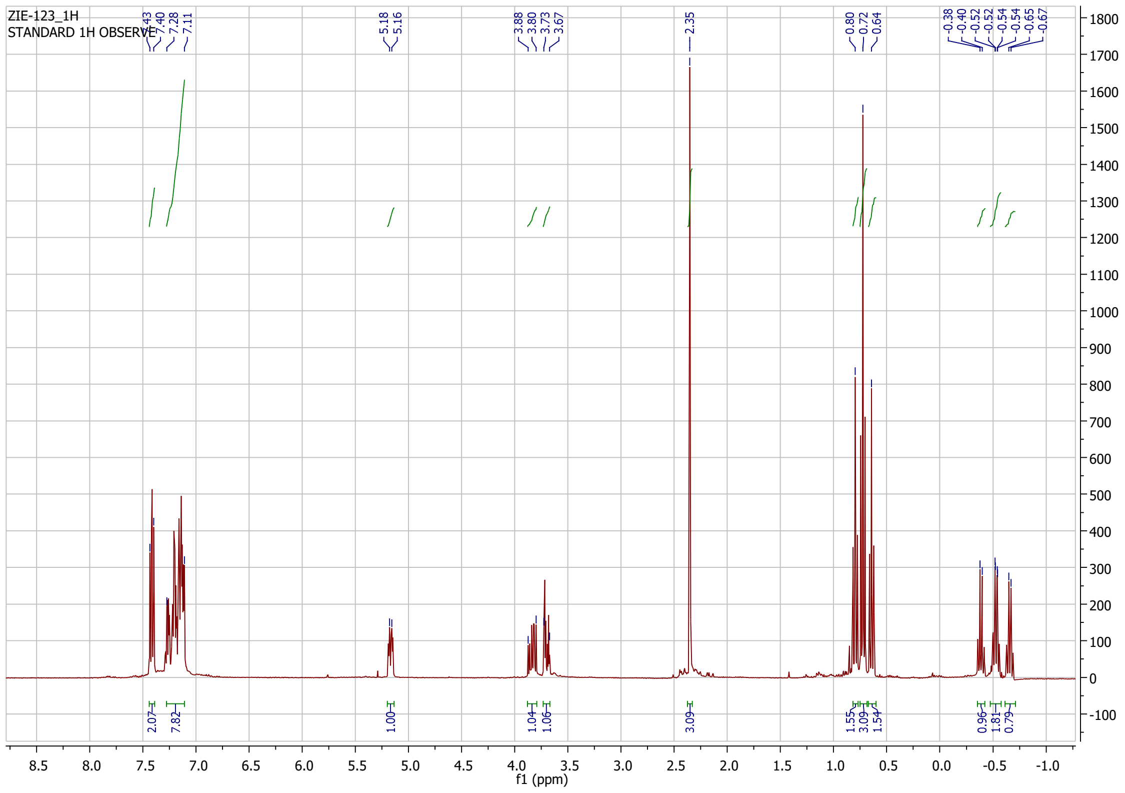

Figure S7. ^{1}H NMR spectrum of the compound **2ba** – hydroalumination product of a *β*-keto sulfone **1a** with Et_3Al (1:1).

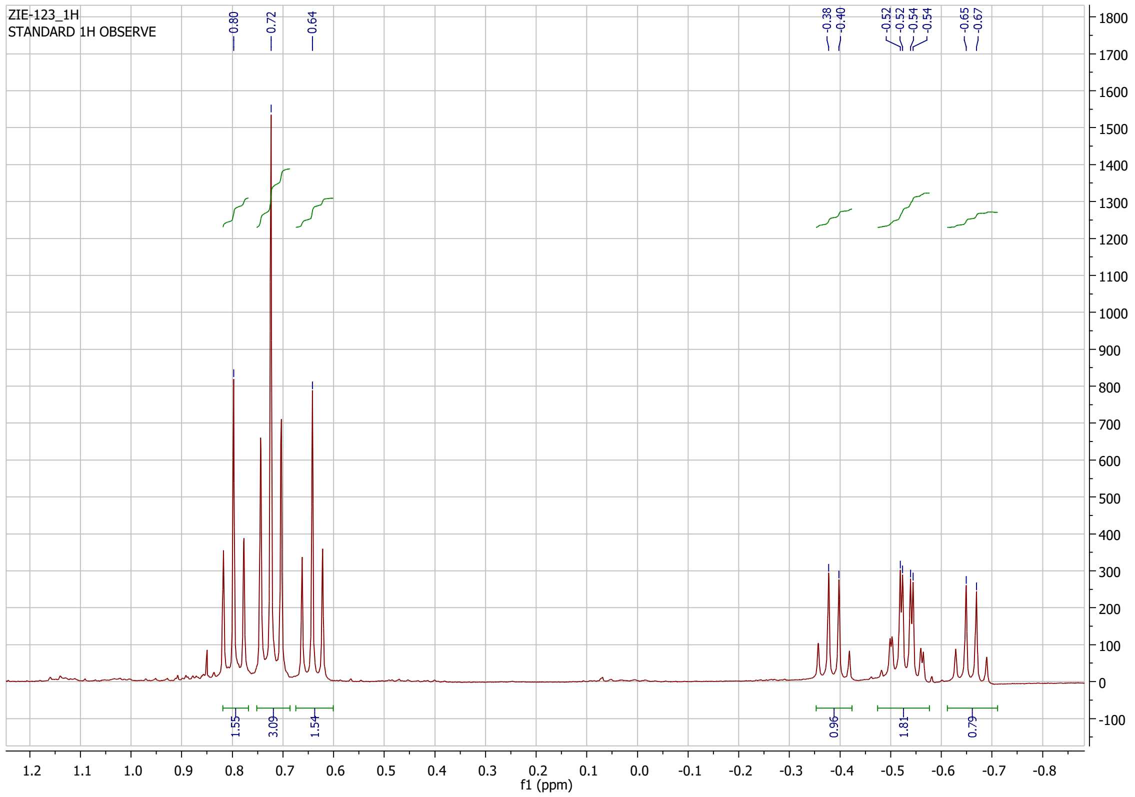

Figure S8. ^{1}H NMR spectrum of the compound **2ba** – expanded part of the spectrum showing EtAl proton signals.

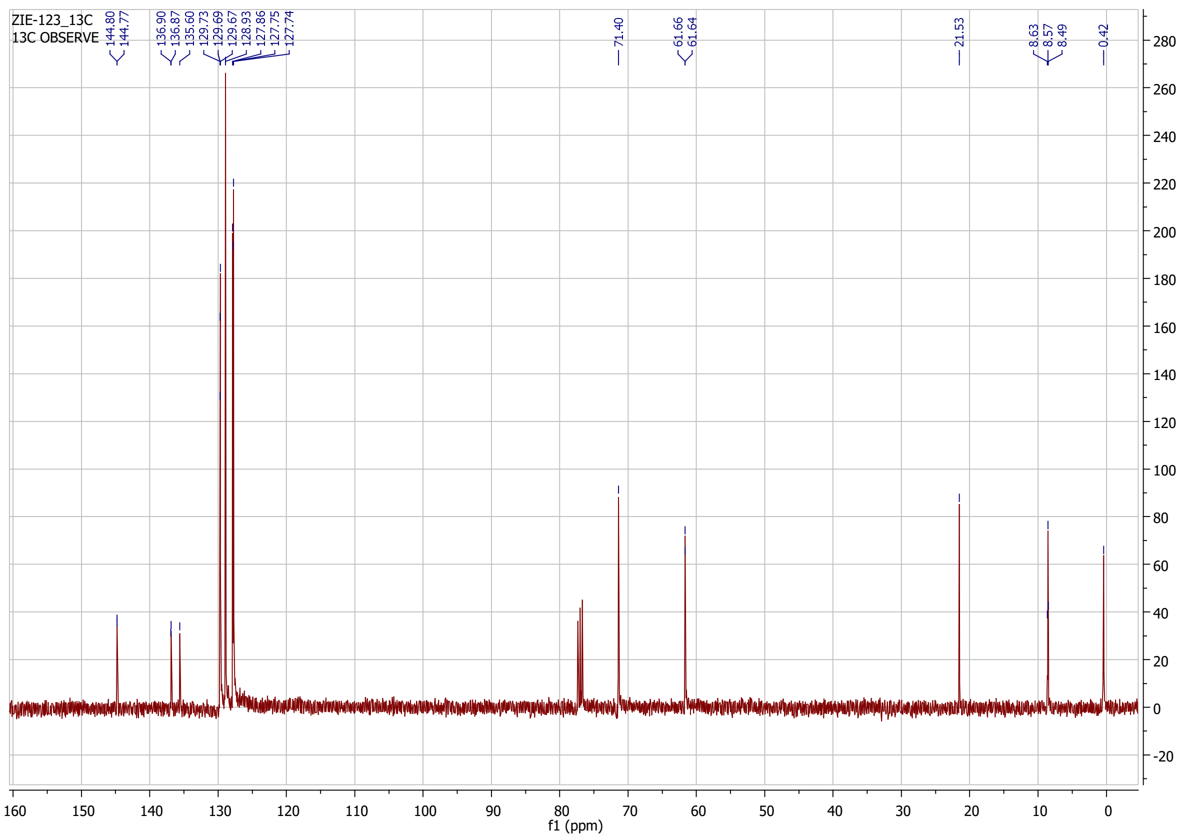

Figure S9. ^{13}C NMR spectrum of the compound **2ba**.

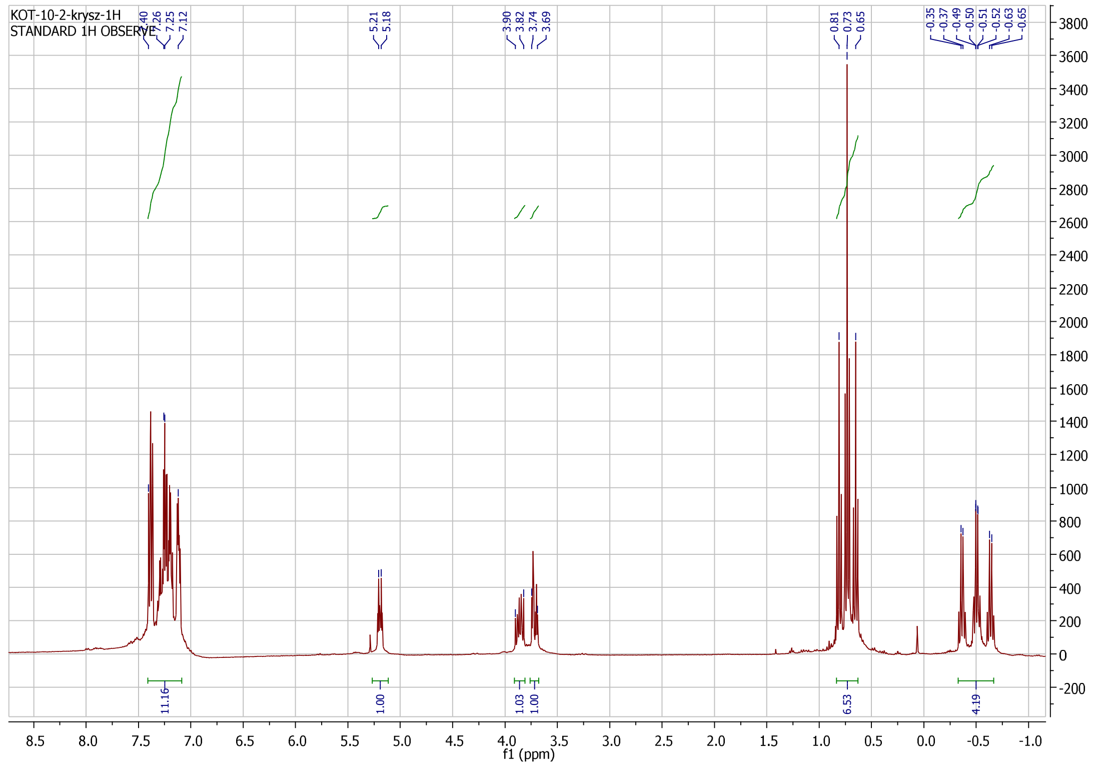

Figure S10. ^{1}H NMR spectrum of the compound **2bb** – hydroalumination product of *β*-keto sulfone **1b** with Et_3Al (1:1).

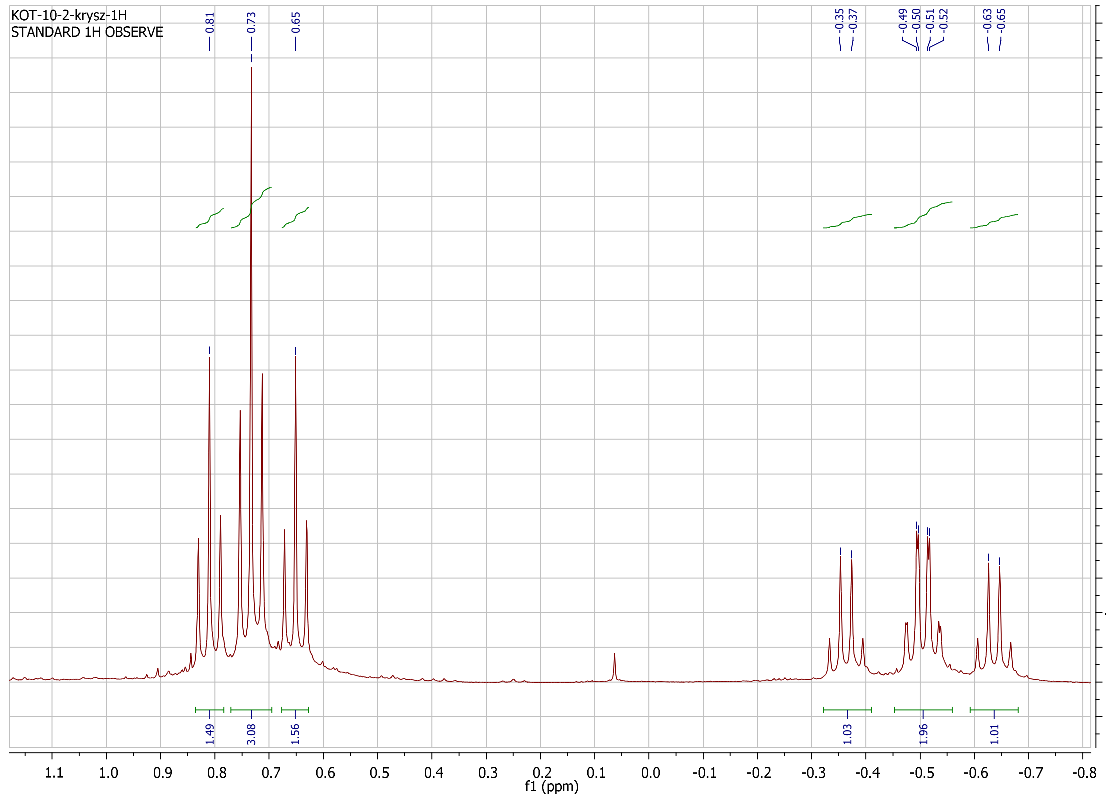

Figure S11. ^{1}H NMR spectrum of the compound **2bb** – expanded part of the spectrum showing EtAl proton signals.

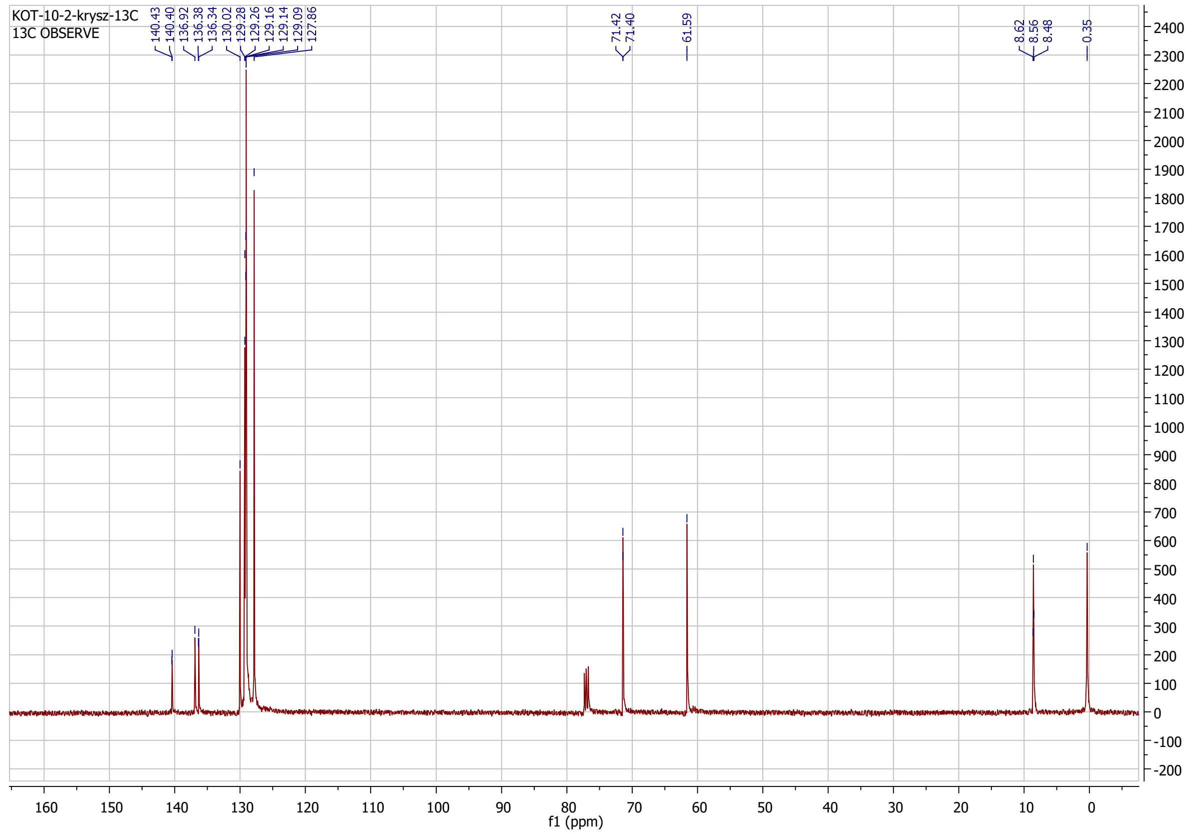

Figure S12. ^{13}C NMR spectrum of the compound **2bb**.

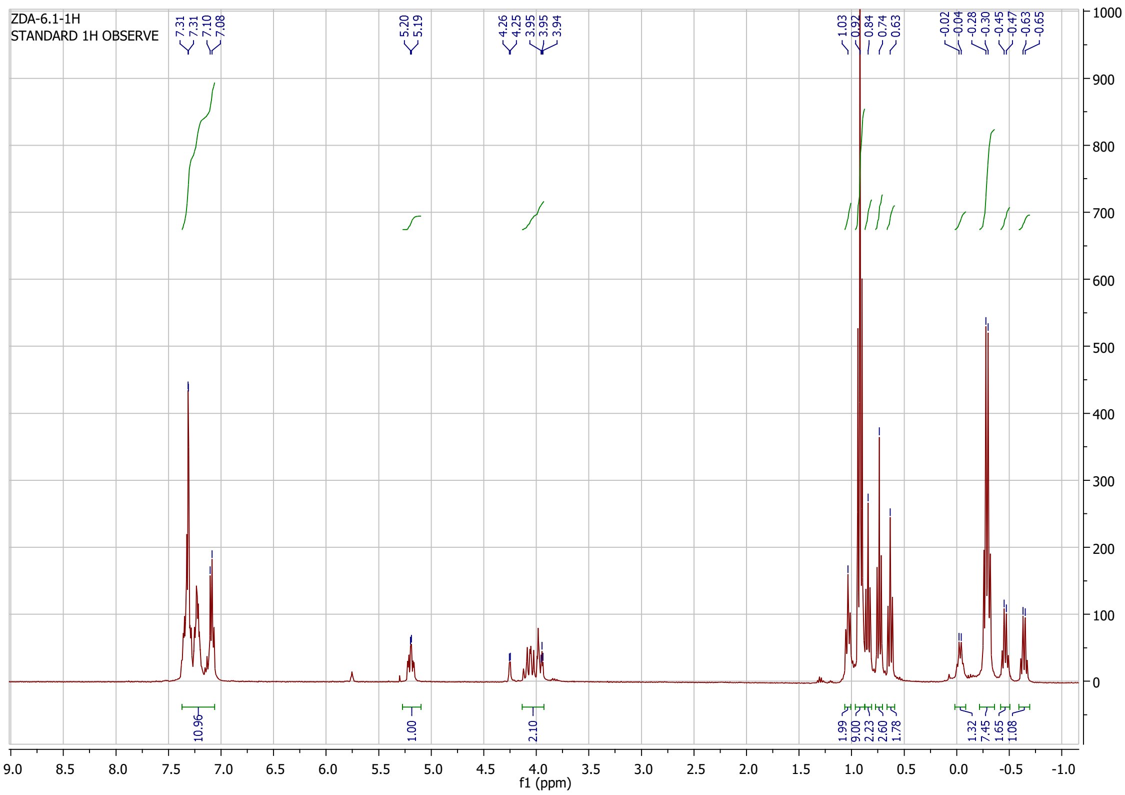

Figure S13. ^{1}H NMR spectrum of the compound **3bb** – hydroalumination product of a *β*-keto sulfone **1b** with Et_3Al (1:2).

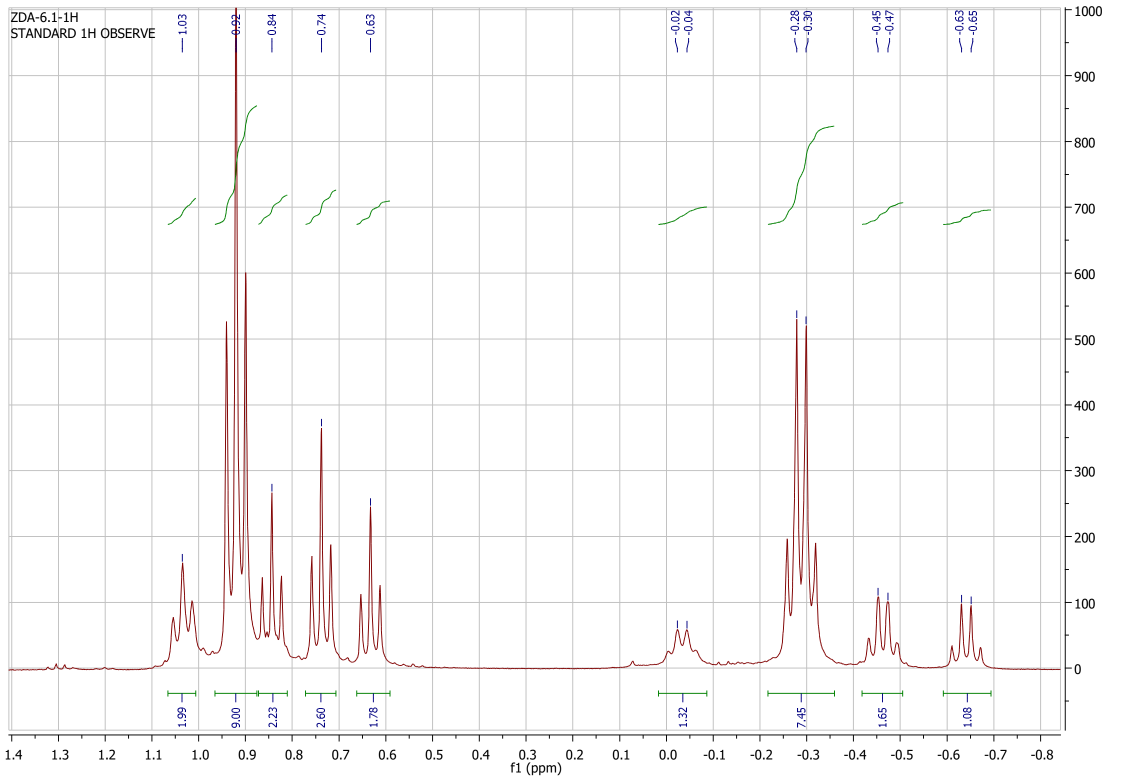

Figure S14. ^{1}H NMR spectrum of the compound **3bb** – expanded part of the spectrum showing EtAl proton signals.

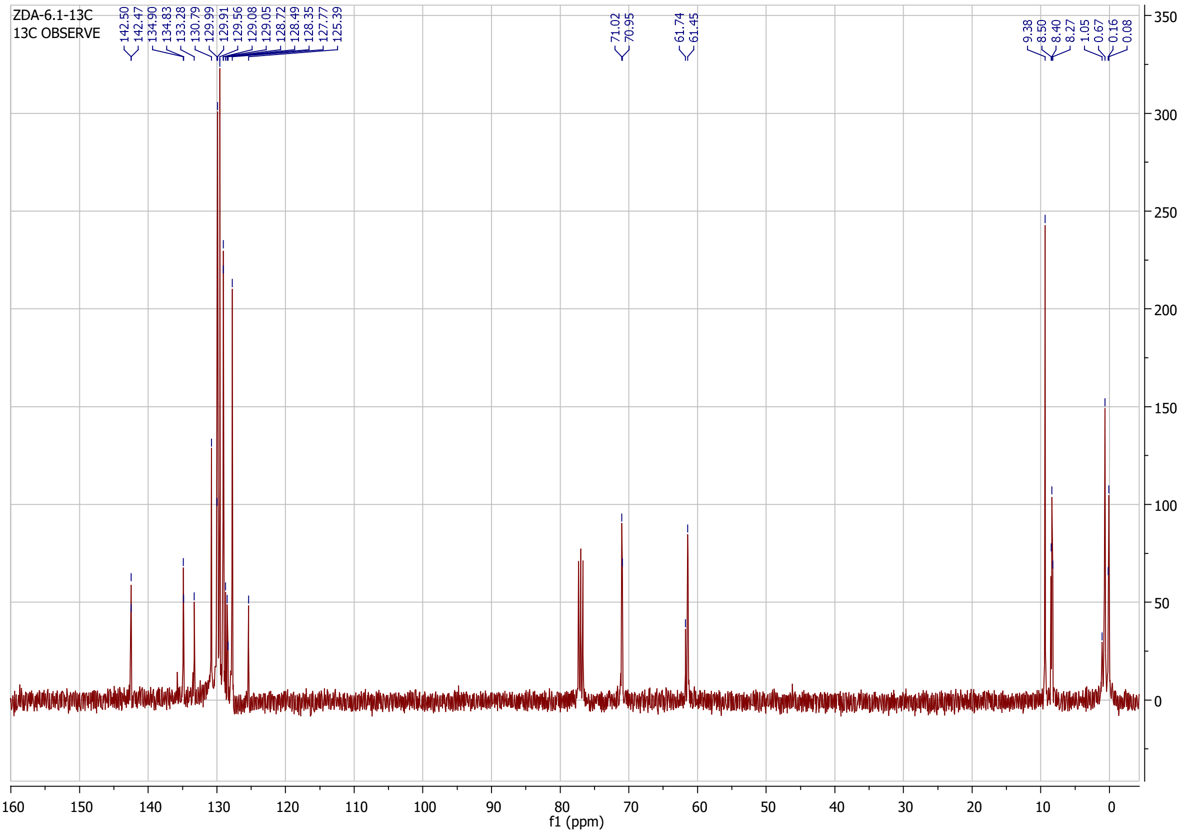

Figure S15. ^{13}C NMR spectrum of the compound **3bb**.

Table S1. Crystal data and data collection parameters for the compounds **2aa** and **2ab**.

	**2aa**	**2ab**
Empirical formula	$C_{46}H_{66}Al_2O_6S_2\cdot 2CH_2Cl_2$	$C_{44}H_{60}Al_2Cl_2O_6S_2\cdot 1.91CH_2Cl_2$
Formula weight	1002.92	1037.63
Temperature (K)	130(2)	130(2)
Wavelength (Å)	0.71073	0.71073
Crystal system	triclinic	triclinic
Space group	P -1	P -1
a(Å)	10.724(2)	10.752(3)
b(Å)	11.645(2)	11.605(3)
c(Å)	12.127(2)	11.846(3)
α(°)	94.927(6)	93.97(1)
β(°)	102.266(7)	102.80(1)
γ(°)	115.243(6)	114.44(1)
V($Å^3$)	501.46	1290.4(6)
Z	2	1
D_{calc}(g cm^{-3})	1.270	1.335
Absorption coefficient (mm^{-1})	0.383	0.488
F(000)	532	545
Crystal size (mm)	0.416 × 0.314 × 0.216	0.270 × 0.261 × 0.212
Θ range for data collection (°)	2.17 to 29.04	2.1867 to 33.0787
Index ranges	$-14 \leq h \leq 14$, $-15 \leq k \leq 15$, $-16 \leq l \leq 16$	$-14 \leq h \leq 14$, $-14 \leq k \leq 14$, $-15 \leq l \leq 15$
Reflections collected	56932	70819
Independent reflections	7004 [R(int) = 0.0519]	7904 [R(int) = 0.0376]
Refinement method	Full-matrix least-squares on F^2	Full–matrix least–squares on F^2
Data / restraints / parameters	7004 / 0 / 290	7904 / 0 / 317
Goodness-of-fit on F^2	1.120	1.041
Final R indices [I>2□(I)]	R_1 = 0.0534, wR_2 = 0.0953	R_1 = 0.0409, wR_2 = 0.0996
R indices (all data)	R_1 = 0.0703, wR_2 =	R_1 = 0.0568, wR_2 = 0.1118

	0.1030	
Max/Min of residual electron density	0.541 and -0.490	0.429 and -0.352

Table SI2. Crystal data and data collection parameters for the compounds **2ba**, **2bb** and **3bb**.

	**2ba**	**2bb**	**3bb**
Empirical formula	$C_{38}H_{50}Al_2O_6S_2$	$C_{36}H_{44}Al_2Cl_2O_6S_2$	$C_{48}H_{74}Al_4Cl_2O_6S_2·0.9CH_2Cl_2$
Formula weight	720.86	761.69	1066.44
Temperature (K)	130(2)	130(2)	110(2)
Wavelength (Å)	0.71073	0.71073	0.71073
Crystal system	triclinic	triclinic	triclinic
Space group	P -1	P -1	P -1
a(Å)	8.115(1)	7.9056(7)	8.359(1)
b(Å)	8.713(1)	8.7706(8)	12.303(2)
c(Å)	15.231(2)	14.975(1)	14.175(2)
α(°)	99.119(6)	78.228(4)	82.222(5)
β(°)	99.465(6)	88.124(4)	85.516(5)
γ(°)	114.254(5)	65.893(4)	75.848(6)
V(Å^3)	937.3(3)	926.3(2)	1399.0(4)
Z	1	1	1
D_{calc}(g cm^{-3})	1.277	1.365	1.266
Absorption coefficient (mm^{-1})	0.233	0.379	0.383
F(000)	384	400	566
Crystal size (mm)	0.432×0.342×0.325	0.523×0.285×0.134	0.525×0.188×0.138
Θ range for data collection (°)	2.6675 to 34.9694	2.60 to 27.50	2.4901 to 26.8221
Index ranges	−12 ≤h ≤12, −13 ≤k ≤13, −23 ≤l ≤ 23	−10 ≤h ≤10, −11 ≤k ≤11, −19 ≤l ≤ 19	−10 ≤h ≤10, −15 ≤k ≤15, −17 ≤l ≤ 17
Reflections collected	63588	36618	38257
Independent reflections	6783 [R(int) = 0.0456]	4260 [R(int) = 0.0372]	5497 [R(int) = 0.0596]

Refinement method	Full-matrix least-squares on F^2	Full-matrix least-squares on F^2	Full-matrix least-squares on F^2
Data / restraints / parameters	6783 / 0 / 220	4260 / 0 / 219	5497 / 33 / 352
Goodness-of-fit on F^2	1.170	1.056	1.045
Final R indices [I>2□(I)]	R_1 = 0.0508, wR_2 = 0.1196	R_1 = 0.0299, wR_2 = 0.0694	R_1 = 0.0367, wR_2 = 0.0785
R indices (all data)	R_1 = 0.0596, wR_2 = 0.1236	R_1 = 0.0350, wR_2 = 0.0728	R_1 = 0.0545, wR_2 = 0.0890
Max/Min of residual electron density	1.011 and -0.451	0.403 and -0.395	0.490 and -0.394
